# A *Gata4* nuclear GFP transcriptional reporter to study endoderm and cardiac development in the mouse

**DOI:** 10.1242/bio.036517

**Published:** 2018-12-15

**Authors:** Claire S. Simon, Lu Zhang, Tao Wu, Weibin Cai, Nestor Saiz, Sonja Nowotschin, Chen-Leng Cai, Anna-Katerina Hadjantonakis

**Affiliations:** 1 Developmental Biology Program, Sloan Kettering Institute, Memorial Sloan Kettering Cancer Center, New York, NY 10065, USA; 2 Department of Developmental and Regenerative Biology, The Mindich Child Health and Development Institute, and The Black Family Stem Cell Institute, Icahn School of Medicine at Mount Sinai, New York, NY 10029, USA; 3 School of Basic Medicine and Clinical Pharmacy, China Pharmaceutical University, Nanjing 211198, China

**Keywords:** Mouse, Gata4, H2B-GFP, Endoderm, Heart, Live imaging

## Abstract

The GATA zinc-finger transcription factor GATA4 is expressed in a variety of tissues during mouse embryonic development and in adult organs. These include the primitive endoderm of the blastocyst, visceral endoderm of the early post-implantation embryo, as well as lateral plate mesoderm, developing heart, liver, lung and gonads. Here, we generate a novel *Gata4* targeted allele used to generate both a *Gata4^H2B-GFP^* transcriptional reporter and a *Gata4^FLAG^* fusion protein to analyse dynamic expression domains. We demonstrate that the *Gata4^H2B-GFP^* transcriptional reporter faithfully recapitulates known sites of *Gata4* mRNA expression and correlates with endogenous GATA4 protein levels. This reporter labels nuclei of *Gata4* expressing cells and is suitable for time-lapse imaging and single cell analyses. As such, this *Gata4^H2B-GFP^* allele will be a useful tool for studying *Gata4* expression and transcriptional regulation.

This article has an associated First Person interview with the first author of the paper.

## Abstract (precis)

**Summary:** Generation of a *Gata4^H2B-GFP^* reporter mouse that labels primitive endoderm, visceral endoderm and cardiac lineages during embryonic development suitable for single cell and live imaging analyses.

## INTRODUCTION

GATA4 is a member of the highly conserved GATA family zinc-finger transcription factors (Viger et al., 2008), which bind to the GATA motif consensus sequence 5-AGATAG-3 in promoter and *cis*-regulatory elements of target genes to activate transcription (Arceci et al., 1993; Merika and Orkin, 1993). The GATA factors can be subdivided into two families based on phylogenetic analysis and their tissue expression profiles; the hematopoietic factors: GATA1/2/3, and the endodermal and cardiac factors: GATA4/5/6 (Crispino and Horwitz, 2017; Peterkin et al., 2005; Lentjes et al., 2016). In humans, *GATA4* mutations are associated with a wide range of congenital heart defects including ventricular septal defects and gonadal anomalies (Garg et al., 2003; Pehlivan et al., 1999; Lourenco et al., 2011; Wang et al., 2011).

In mice, *Gata4* plays an important role in the development of extra-embryonic endoderm. During pre-implantation development, from embryonic day (E) ∼3.5 to ∼E4.5, the inner cell mass (ICM) of the blastocyst gives rise to the pluripotent epiblast (Epi) and the extra-embryonic primitive endoderm (PrE) (Rossant, 2016; Chazaud and Yamanaka, 2016). While other PrE markers GATA6, PDGFRA, SOX17 are expressed to some extent in uncommitted ICM cells, GATA4 is the earliest known PrE-specific marker (Artus et al., 2011; Plusa et al., 2008; Kurimoto et al., 2006; Arceci et al., 1993; Niakan et al., 2010; Morris et al., 2010; Chazaud et al., 2006; Ohnishi et al., 2014), making it a prime candidate reporter for the identification of PrE cells as they emerge in real-time. Overexpression of either GATA4 or GATA6 is sufficient to induce differentiation of pluripotent embryonic stem (ES) cells to extra-embryonic endoderm like (XEN) cells (Fujikura et al., 2002; Schroter et al., 2015; Morrisey et al., 1996; Shimosato et al., 2007; Niakan et al., 2013), although *Gata4* is dispensable for PrE specification *in vivo* (Molkentin et al., 1997; Kuo et al., 1997).

*Gata4* is functionally required later in the extra-embryonic endoderm at early post-implantation stages, where it is expressed in the parietal (ParE) and visceral (VE) endoderm (Heikinheimo et al., 1994; Arceci et al., 1993; Cai et al., 2008). A third of *Gata4^−/−^* embryos fail to gastrulate, causing early lethality at ∼E7.5 (Molkentin et al., 1997). The remaining two thirds of *Gata4^−/−^* embryos arrest at ∼E9.0 due to a failure in ventral closure leading to defective heart morphogenesis (Molkentin et al., 1997; Kuo et al., 1997). These phenotypes can be rescued with wild-type extra-embryonic tissues (Narita et al., 1997; Watt et al., 2004, 2007), indicating that abnormal VE development is the primary defect in both classes of *Gata4^−/−^* embryos. In support of *Gata4* playing a key role in VE development, *Gata4^−/−^* ES cells fail to efficiently differentiate into VE-like cells (Soudais et al., 1995).

*Gata4* also plays essential roles in cardiac development (Pikkarainen et al., 2004; Peterkin et al., 2005; Zhao et al., 2008). Tetraploid complementation with *Gata4*^−/−^ ES cells produce embryos with abnormal heart tube looping and thin-walled myocardium (Watt et al., 2007, 2004). Disruption of *Gata4* function in the heart by either tissue-specific ablation, impaired FOG2 interaction, or substitution with *Gata6,* all result in severe cardiac abnormalities (Zeisberg et al., 2005; Oka et al., 2006; Crispino et al., 2001; Rojas et al., 2008; Rivera-Feliciano et al., 2006; Borok et al., 2016). GATA4 therefore is considered a key regulator of cardiogenesis, and *Gata4* mRNA is broadly expressed throughout cardiac development (Auda-Boucher et al., 2000; Arceci et al., 1993). During gastrulation, *Gata4* transcripts are detected in the cardiac and lateral plate mesoderm (Heikinheimo et al., 1994; Rojas et al., 2005; Schachterle et al., 2012). *Gata4* expression continues in the primitive heart tube from E8.0 in both the endocardium and myocardium, and persists in cardiomyocytes throughout embryonic development and into adulthood (Heikinheimo et al., 1994; Morrisey et al., 1996; Schachterle et al., 2012). In addition to its roles in VE and cardiac morphogenesis, *Gata4* expression is also required for proper development of the ovary, testes, pancreas, lung and liver (Kyrönlahti et al., 2011a,b; Watt et al., 2007).

Due to the pivotal roles of *Gata4* in a variety of tissues, visualising the dynamic spatial and temporal expression domains is of great importance. Tissue-specific sites of *Gata4* expression have been recapitulated with *cis*-regulatory elements driving transgenic LacZ reporters, for example in the lateral mesoderm (G2 enhancer), cardiac lineage (G9 enhancer) and the endoderm (G4 and G8 enhancers) (Rojas et al., 2005, 2009, 2010; Schachterle et al., 2012). Endogenous sites *Gata4* expression can be replicated with *rGata4p-[5kb]GFP* and *rGata4p-[5kb]RFP* transgenic reporters which contain a 5*kb* fragment of the rat *Gata4* proximal promoter (Pilon et al., 2008; Mazaud Guittot et al., 2007)*.* However, single-cell resolution image analysis of *rGata4p-[5kb]GFP* and *rGata4p-[5kb]RFP* is challenging due to the cytoplasmic localisation of the fluorescence proteins used in these reporters.

Here, to circumvent transgenic site-specific and copy-number integration effects on reporter expression, and to conserve all *cis*-regulatory sequences, we generated a novel nuclear localised fluorescent reporter and a FLAG fusion targeted to the endogenous *Gata4* locus. The transcriptional reporter, *Gata4^H2B-GFP^*, faithfully recapitulated known sites of *Gata4* expression and strongly correlated with GATA4 protein levels. GFP expression was robustly observed in the endoderm lineages of the PrE, VE and midgut. In addition, GFP was also expressed in mesodermal cell types; early extra-embryonic mesoderm, cardiac lineage, lateral plate mesoderm, septum transversum, and then later in the lung and hepatic mesenchyme. This reporter brightly labelled nuclei of *Gata4* expressing cells and was suitable for time-lapse imaging and single-cell resolution analysis. In addition, Cre recombination of the Gata4*^H2B-GFP^* allele generates a *Gata4^FLAG^* allele encoding a fusion protein for use in biochemical studies. As such, the *Gata4^H2B-GFP^* and *Gata4^FLAG^* alleles, represents useful tools for identifying novel sites of *Gata4* expression and yield insights into how this key transcription factor is regulated and functions during embryonic development and tissue morphogenesis.

## RESULTS AND DISCUSSION

### Generation of a *Gata4*^H2B-GFP^ reporter

To follow transcriptional activity of *Gata4* in mouse embryonic development, we generated a bright nuclear localised fluorescent reporter that would be suitable for live imaging and for single-cell resolution analysis. For this, we used a histone-fused GFP protein H2B-GFP (Kanda et al., 1998; Hadjantonakis and Papaioannou, 2004). To circumvent site-specific integration effects of transgenes on reporter expression, and to conserve all *cis*-regulatory sequences we engineered a targeted allele at the endogenous *Gata4* locus.

**Fig. 1.**
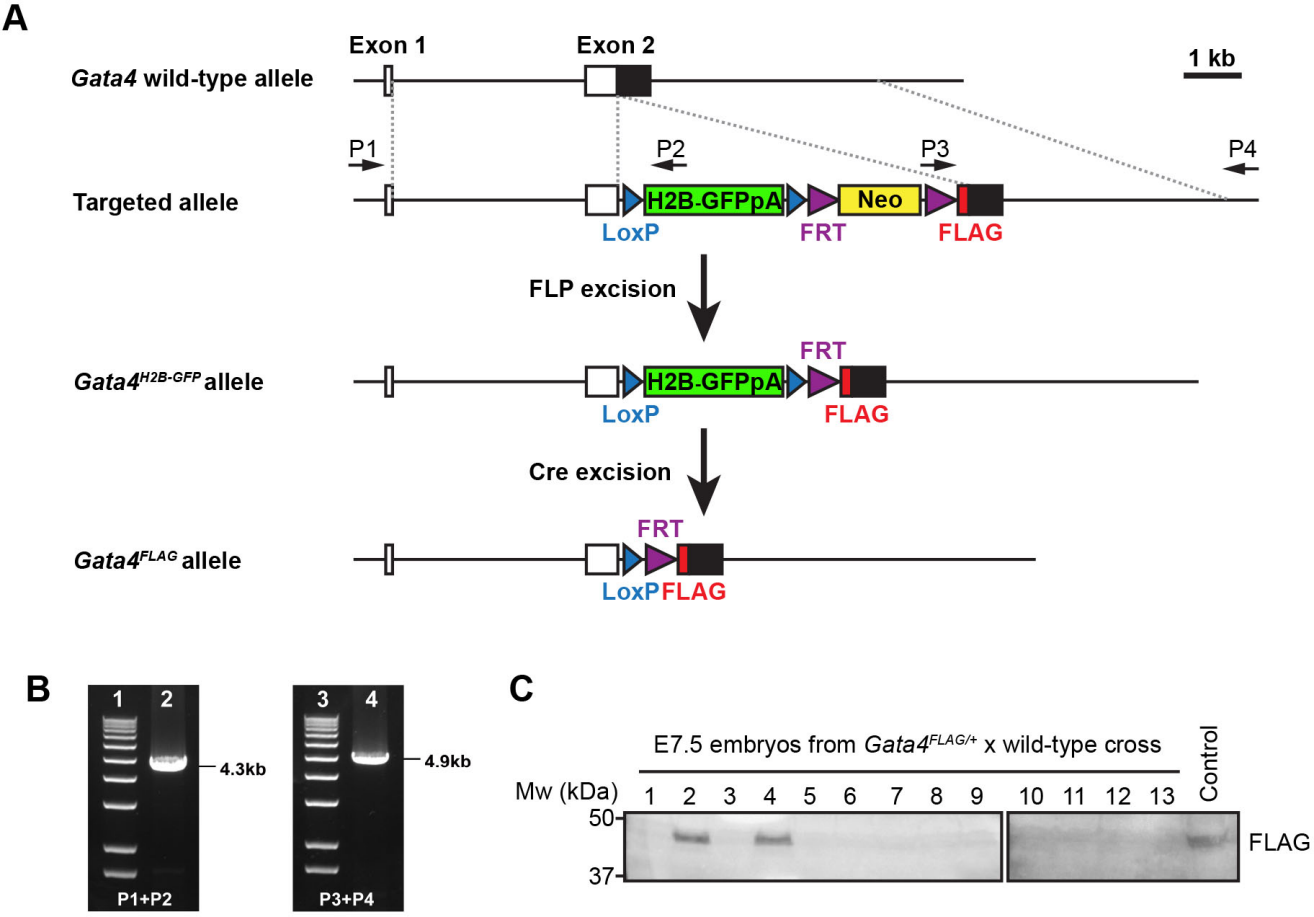
**Generation of *Gata4^H2B-GFP/+^* targeted mouse model.** (A) The cassette LoxP-H2B-GFP-4XPloyA-LoxP-FRT-Neo-FRT-3XFLAG was inserted into the start codon of the mouse *Gata4* locus through homologous recombination. The Neo cassette was flanked by two FRT sites and removed by crossing to Flippase mice to generate the *Gata4^H2B-GFP^* allele. Cre excision of H2B-GFP generates GATA4-FLAG N-terminal protein fusion *Gata4^FLAG^* allele. (B) The targeted ES cells were confirmed by long-range PCR amplification of two fragments using primers P1 and P2, P3 and P4, respectively. Primers P1 and P2 produced a 4.3* kb* band. Primers P3 and P4 produced a 4.9* kb* band. (C) Western blot of individual E7.5 embryo littermates from a *Gata4^FLAG/+^*×wild-type mating. The anti-FLAG antibody cross reacts with GATA4-FLAG in *Gata4^FLAG/+^* embryos. Total protein lysis of an ear from a *Gata4^FLAG/+^* adult male was used as a positive control.

The *Gata4*^H2B-GFP/+^ knock-in mouse model was generated by inserting a *loxP*-flanked *H2B-GFP-4×PolyA* cassette, followed by a *FRT*-flanked *Neo* selection cassette, and a *3×FLAG* tag into the endogenous ATG start site of *Gata4* exon 2. This was achieved by homologous recombination in mouse embryonic stem (ES) cells (Fig. 1A). Correctly targeted ES cells were screened by long-range PCR (Fig. 1B), and were then used to generate germline chimeric mice. The *FRT* flanked *Neo* cassette was excised *in vivo* by crossing with a ubiquitously expressing FLP mouse line to generate the *Gata4^H2B-GFP^* allele. The *H2B-GFP-4×PolyA* sequence upstream of the *Gata4* coding sequence renders the *Gata4^H2B-GFP^* a null allele, and no homozygous mice could be recovered at weaning age (Table 1).

**Table 1.**
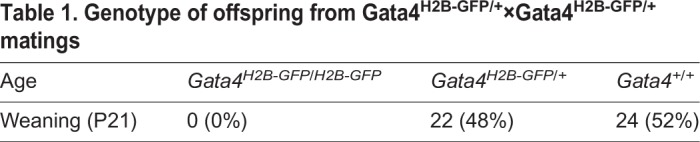
**Genotype of offspring from Gata4^H2B-GFP/+^×Gata4^H2B-GFP/+^ matings**

Cre-mediated excision of the *LoxP*-flanked *H2B-GFP-4×PolyA* generated a *Gata4^FLAG^* allele that encodes an *N*-terminal 3×FLAG tagged GATA4 fusion protein. Western blot of individual E7.5 littermate embryos from a *Gata4^FLAG/+^* and wild-type mating demonstrated GATA4-FLAG protein expression in those embryos that presumably inherited the *Gata4^Flag^* allele. The GATA4-FLAG fusion protein had an approximate molecular weight of 47 kDa, in agreement with its predicted size. *Gata4^FLAG^* heterozygous and homozygous mice were viable and fertile (Table S1). This suggests that the GATA4-FLAG protein fusion does not markedly disrupt GATA4 protein function, due to the absence of early embryonic lethality that is seen in *Gata4* null mice.

### Primitive endoderm cells express *Gata4^H2B-GFP^* reporter at the blastocyst stage

Uncommitted ICM cells of the blastocyst co-express the transcription factors NANOG and GATA6, a pattern that is gradually and asynchronously resolved as cells become specified between the 32–100 cell stages to NANOG+ Epi cells and GATA6+ PrE cells (Saiz et al., 2016b; Plusa et al., 2008; Chazaud et al., 2006). PrE specification and lineage maturation follows a sequential, hierarchical activation of the PrE lineage-specific markers; GATA6, PDGFRA, SOX17, GATA4 and SOX7 (Plusa et al., 2008; Arceci et al., 1993; Artus et al., 2011; Kurimoto et al., 2006; Niakan et al., 2010; Morris et al., 2010; Chazaud et al., 2006). Mature PrE cells express GATA4 from ∼58–63 cell stage blastocyst (Plusa et al., 2008).

**Fig. 2.**
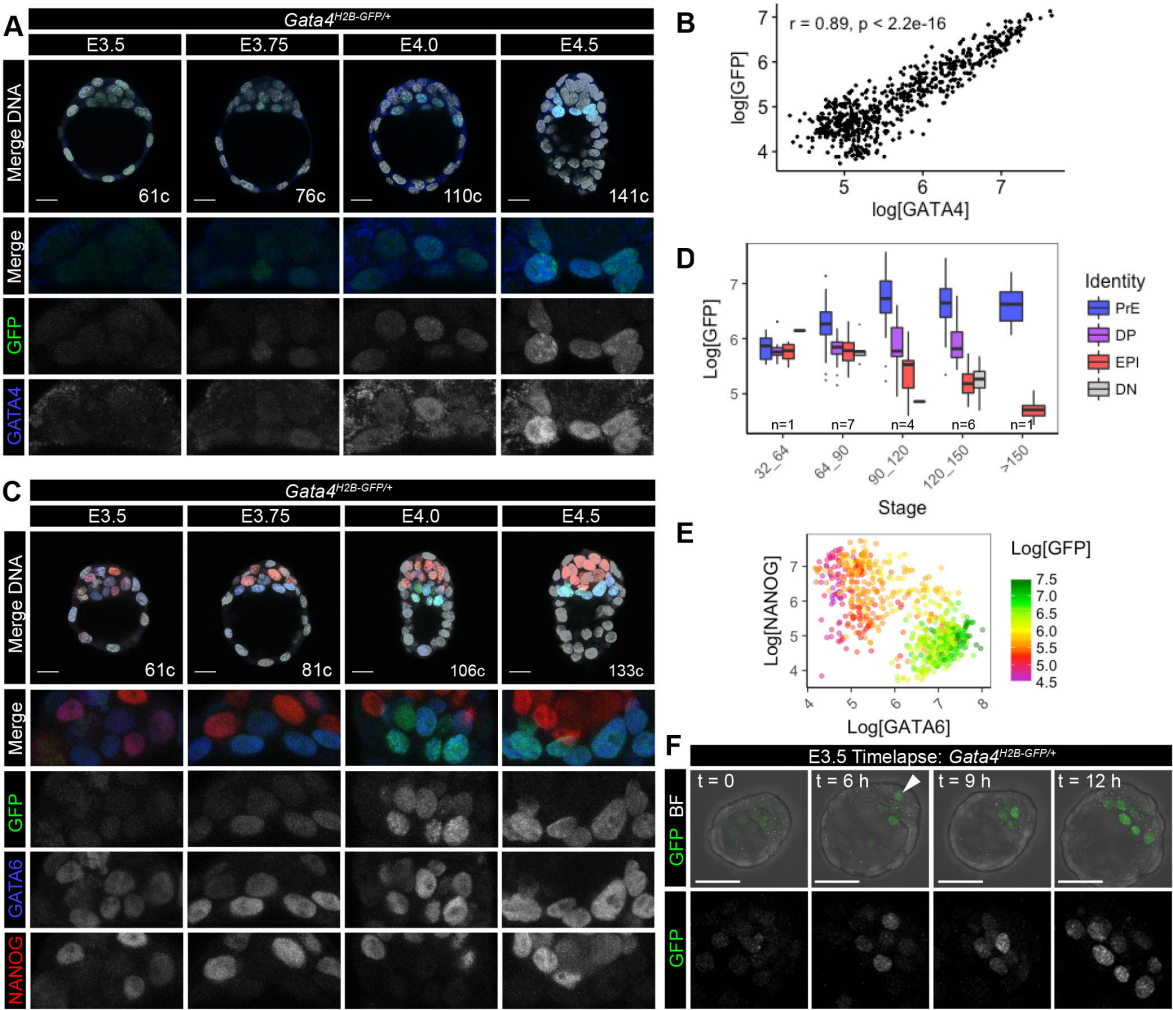
***Gata4^H2B-GFP^* expression marks primitive endoderm during pre-implantation development.** (A) Representative images showing 10 μm maximum intensity projections of confocal z-stack images of fixed *Gata4^H2B-GFP/+^* embryos immunostained for GATA4 (rabbit antibody) at E3.5–E4.5 stages of pre-implantation development. GFP signal was detected directly and nuclei were stained with Hoechst. Quantification shown in B and Fig. S1A. (B) Scatter plot showing the nuclear fluorescence intensity of GFP and endogenous GATA4 protein within individual ICM cells of *Gata4^H2B-GFP/+^* embryos (*n*=20) between E3.5–E4.5 stages. Pearson correlation coefficient (r) and *P* values are shown in the graph. (C) Representative images showing 10 μm maximum intensity projections of confocal z-stack images of *Gata4^H2B-GFP/+^* embryos fixed and immunostained with GFP, GATA6 and NANOG at E3.5–E4.5 stages of pre-implantation development. Nuclei were stained with Hoechst. Quantification and lineage allocation shown in D, E and Fig. S1B. (D) Boxplots showing the GFP nuclear fluorescence intensity in primitive endoderm (PrE, GATA6+, blue), double positive (DP, GATA6+NANOG+, purple), epiblast (EPI, NANOG+, red) and double negative (DN, GATA6−NANOG−, grey) ICM cells of fixed *Gata4^H2B-GFP/+^* embryos (*n*=19). (E) Scatter plot showing the relative nuclear fluorescence intensity of NANOG and GATA6, coloured according to GFP nuclear intensity within individual ICM cells of fixed *Gata4^H2B-GFP/+^* embryos (*n*=19 embryos). (F) Still images of time-lapse microscopy of E3.5 *Gata4^H2B-GFP/+^* embryos with GFP overlaid on bright field (BF) or GFP alone. GFP+ cells emerge from a deep position (arrow) within the ICM and sort onto the surface of the blastocoel cavity. Blastocyst embryo stages represented by cell (c) number. Fixed embryo scale bars: 20 μm; time-lapse scale bars: 50 μm.

To determine when and where the *Gata4^H2B-GFP^* reporter is expressed during pre-implantation development, we directly visualised GFP and stained for GATA4 protein in fixed heterozygous embryos between early-, (32–64 cells), mid- (64–100 cells) and late- (>100 cells) blastocyst stages by laser scanning confocal microscopy (Fig. 2A). Nuclear fluorescent intensities were quantified using a MATLAB-based nuclear segmentation algorithm, MINS (Lou et al., 2014). The expression of the *Gata4^H2B-GFP^* reporter was tightly correlated with GATA4 antibody staining in the ICM across pre-implantation development (r=0.89; Fig. 2B, Fig. S1A). Although occasionally we observed GFP positive cells that did not show nuclear GATA4 staining (Fig. 2A), likely due to H2B-GFP remaining bound to chromatin in mitotic cells or possibly independent regulation of *Gata4* transcription and GATA4 protein levels. *Gata4^H2B-GFP^* expression was observed in ∼62 cell stage embryos that had specified few GATA4+ PrE cells and levels increased over-time within the PrE (Fig. S1A). By the late blastocyst stage (>100 cells) *Gata4^H2B-GFP^* expression was spatially restricted to the PrE epithelial layer lining the blastocyst cavity (Fig. 2A). Similar results were also seen using an alternative GATA4 antibody and anti-GFP antibody (Fig. S1B)

To confirm the association of *Gata4^H2B-GFP^* expression to the PrE, we compared GFP levels between ICM lineages in NANOG and GATA6 stained embryos. Cell identities were assigned as previously described (Saiz et al., 2016b,a), as either PrE (GATA6+), EPI (NANOG+), double positive (DP, NANOG+GATA6+) or double negative (DN, NANOG–GATA6–) (Fig. 2C, Fig. S1C). GFP levels were enriched in PrE (GATA6+NANOG−) cells, and increased in expression in later stage embryos (>90 cells, Fig. 2D,E). *Gata4^H2B-GFP^* expressing cells also co-stained for the PrE markers SOX7 and SOX17 (Fig. S1D,E).

To assess the dynamics of the *Gata4^H2B-GFP^* reporter, we performed time-lapse imaging of pre-implantation embryos. Mid-blastocyst stage *Gata4^H2B-GFP^*^/+^ embryos (∼E3.5) were imaged for 16 h. The *Gata4^H2B-GFP^* reporter was sufficiently bright to follow the trajectory of PrE cells emerging within the ICM to the surface of the blastocoel cavity (Fig. 2F, arrow and Movie 1). To experimentally estimate the half-life of *Gata4^H2B-GFP^* we treated late blastocyst stage (∼E4.0) embryos for 12 h with low levels of cycloheximide (10 μM) to partially inhibit protein translation (Fig. S2A and Movies 2 and 3). The half-life of the exponential decay rate of H2B-GFP under cycloheximide treatment was approximately 5.5 h (Fig. S2B,C), similar to the 6 h half-life observed for *Nanog:H2B-GFP* (Xenopoulos et al., 2015). Together, these data demonstrate the *Gata4^H2B-GFP^* reporter recapitulates endogenous gene expression, faithfully marking the PrE lineage, and can be used to visualise PrE emergence in live embryos.

### Early post-implantation expression of *Gata4^H2B-GFP^* in the visceral endoderm and mesoderm

**Fig. 3.**
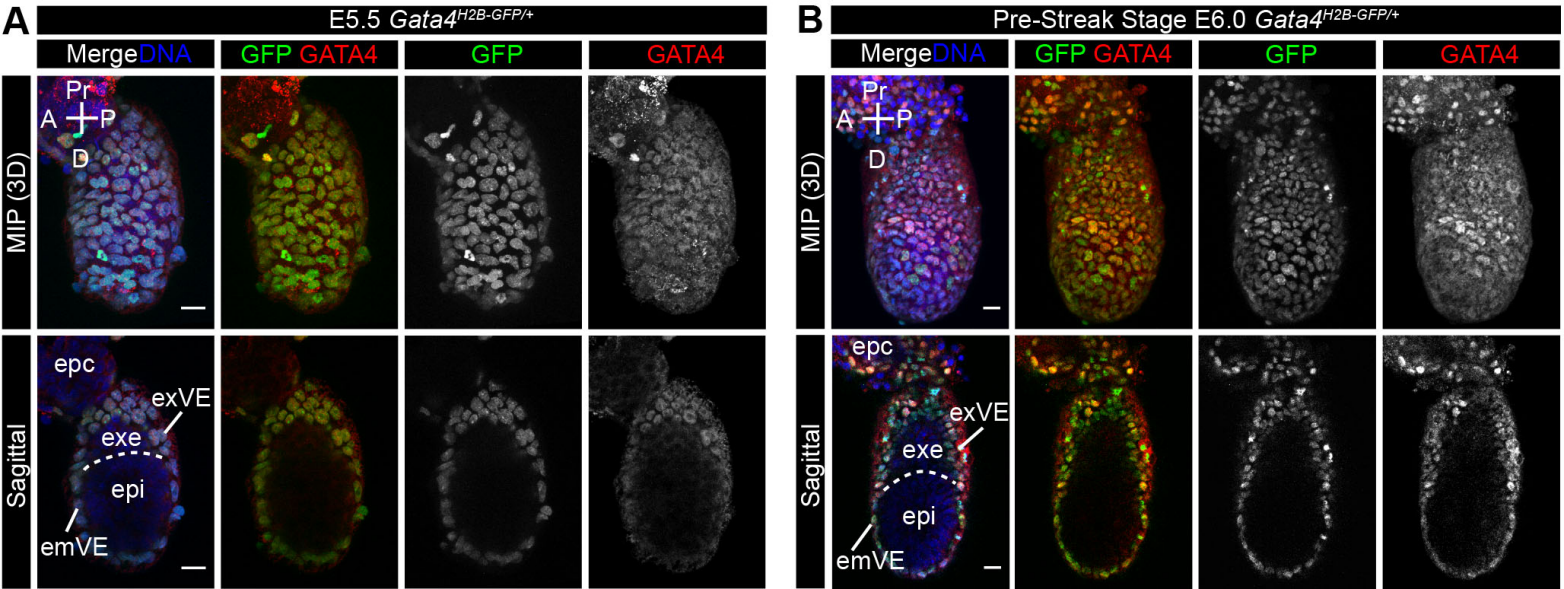
**The *Gata4^H2B-GFP^* reporter is expressed in the visceral endoderm at early post-implantation stages.** (A–B) Representative confocal images of fixed whole-mount *Gata4^H2B-GFP/+^* embryos immunostained for endogenous GATA4 protein at E5.5 (A) and E6.0 (B) post-implantation stages prior to gastrulation. Upper panels show maximum intensity projection (MIP) and lower panels show sagittal views of projected 10um z-stack (A) or single optical plane (B). Nuclei are stained with Hoechst and GFP was visualised directly. GFP and GATA4 are co-localised in extra-embryonic visceral endoderm (exVE) and embryonic visceral endoderm (emVE) but absent in extra-embryonic ectoderm (exe) and epiblast (epi). Scale bars: 20 μm. Ectoplacental cone (EPC), anterior (A), posterior (P), proximal (Pr), distal (D).

The PrE lineage gives rise to the ParE and VE populations of the post-implantation embryo, which both express *Gata4* transcripts (Heikinheimo et al., 1994; Arceci et al., 1993; Morrisey et al., 1998). To determine the expression of *Gata4^H2B-GFP^* in these extra-embryonic lineages at early post-implantation stages, we directly visualised GFP expression and stained for endogenous GATA4 protein in E5.5 and E6.0 *Gata4^H2B-GFP/+^* heterozygous embryos. At E5.5 and at E6.0, prior to overt primitive streak formation (pre-streak; PS stage), GATA4 protein was expressed throughout the embryonic visceral endoderm (emVE) and extra-embryonic visceral endoderm (exVE) (Cai et al., 2008), where it co-localised with GFP expression (Fig. 3A,B). At both E5.5 and E6.0 GFP expression was absent from the epiblast and extra-embryonic ectoderm (ExE) (Fig. 3A,B). Together, these data demonstrate that the *Gata4^H2B-GFP^* allele faithfully recapitulates previously described expression domains of *Gata4* in the VE prior to gastrulation (Cai et al., 2008; Morrisey et al., 1998; Heikinheimo et al., 1994).

At gastrulation (E6.5–E7.25), cells of the proximal-posterior epiblast undergo an epithelial-to-mesenchymal transition (EMT) in the primitive streak region to form the mesoderm and definitive endoderm (DE), and migrate between the epiblast and VE epithelial layers (Arnold and Robertson, 2009; Viotti et al., 2014a). By early bud stage (E7.5) DE cells intercalate into and disperse the overlying emVE cell layer (Kwon et al., 2008; Viotti et al., 2014b).

**Fig. 4.**
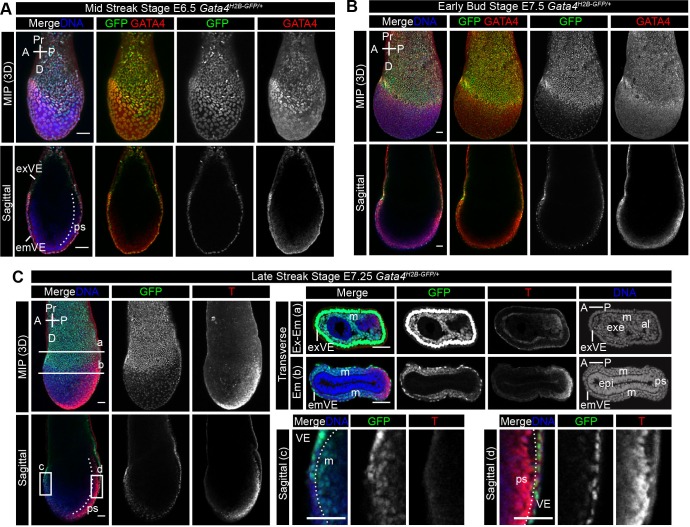
***Gata4^H2B-GFP/+^* reporter expression marks the visceral endoderm, cardiac and lateral plate mesoderm at gastrulation stages.** (A–B) Representative confocal images of fixed whole-mount *Gata4^H2B-GFP/+^* embryos immunostained for endogenous GATA4 protein at E6.5 (A) and E7.5 (B) post-implantation stages during gastrulation. Upper panels show maximum intensity projection (MIP) and lower panels show sagittal views of a single optical plane (B). Nuclei are stained with Hoechst and GFP was visualised directly. (A) GFP expression throughout the embryonic and extra-embryonic visceral endoderm (emVE and exVE) and co-localisation with endogenous GATA4 protein. (B) After definitive endoderm intercalation and emVE dispersal, GFP+ emVE cells remain dispersed over the epiblast. Upper panels show maximum intensity projection (MIP) and lower panels show sagittal views of a single optical plane. (C) Representative confocal images of fixed whole-mount *Gata4^H2B-GFP/+^* embryos immunostained for the primitive streak (ps) and nascent mesoderm marker BRACHYURY (T) at E7.25. Upper panels show maximum intensity projection (MIP) and lower panels show sagittal views of a single optical plane. (a,b) Transverse section at the positions indicated on MIP images. (a) Transverse section though the extra-embryonic (Ex-Em) region showing high levels of GFP fluorescence in the exVE, with lower levels of GFP fluorescence in extra-embryonic mesoderm. (b) Transverse section though the embryonic (Em) region showing lower levels of GFP fluorescence in the embryonic mesoderm, likely cardiac and lateral plate mesoderm, and emVE. Boxed regions on the sagittal view (c) show higher magnification of anterior region with GFP+ cells in the mesoderm layer, and (d) higher magnification of the posterior primitive streak region with GFP expression only present in the emVE. Scale bars: 50 μm. Anterior (A), posterior (P), proximal (Pr), distal (D), mesoderm (mes, or m), epiblast (epi), extra-embryonic ectoderm (exe), allantois (al).

To assess *Gata4^H2B-GFP^* expression during gastrulation we visualised GFP and GATA4 protein in E6.25–E7.75 *Gata4^H2B-GFP/+^* embryos. At the early- and mid-streak stage (E6.25–E6.5) GFP is expressed throughout emVE and exVE cells, where it is largely co-expressed with GATA4 protein in both the exVE and emVE (Fig. 4A, Fig. S3A). The GFP+ emVE cells overlying the anterior region of the epiblast expressed the highest levels of GATA4, however, in the posterior region of the emVE, we observed GFP positive cells that did not show nuclear GATA4 staining (Fig. 4A). The broader domain of *Gata4^H2B-GFP^* expression may be due independent regulation of transcript and protein levels, or the perdurance of the GFP protein. At the early bud stage and early head fold stage (E7.5–E7.75), after emVE dispersal, the sparse emVE cells overlying the epiblast expressed GFP (Fig. 4B, Fig. S3B). Together, these data suggest that at gastrulation stages *Gata4^H2B-GFP^* marks the entire VE (both exVE and emVE), both before and after emVE dispersal.

At the early bud stage and early head fold stage (E7.5–E7.75), GFP+GATA4+ cells were observed in the mesoderm layer (Fig. 4B, Fig. S3B). To better identify *Gata4^H2B-GFP^* reporter expression within cells of the mesoderm, we stained *Gata4^H2B-GFP/+^* late steak (∼E7.25) embryos for BRACHYURY (T) to mark the primitive streak and nascent mesoderm (Fig. 4C). Transverse cryo-sections through the extra-embryonic region showed high levels of GFP expression in the exVE, and lower GFP levels in the extra-embryonic mesoderm (Fig. 4Ca), in agreement with known sites of GATA4 expression in extra-embryonic endoderm and mesoderm (Nemer and Nemer, 2003).

Transverse sections through the primitive streak revealed GFP expression in the emVE, and the anterior and lateral migrating mesoderm layer (Fig. 4Cb), likely representing cardiac and lateral plate mesoderm (Rojas et al., 2005; Schachterle et al., 2012; Zhao et al., 2008). T expression in the nascent mesoderm was restricted to the posterior region of the embryo, and did not coincide with GFP expression (Fig. 4Cb). Reciprocal expression of T and GFP in the posterior and anterior mesoderm was also seen in sagittal single optical views of whole-mount embryos (Fig. 4Cc,d). These data suggest that as cardiac mesoderm and lateral mesoderm cells migrate away from the streak they downregulate *T* and activate *Gata4* expression, similar to the pattern of T/*Gata6* at this stage (Freyer et al., 2015).

### *Gata4^H2B-GFP^* expression at mid-gestation

**Fig. 5.**
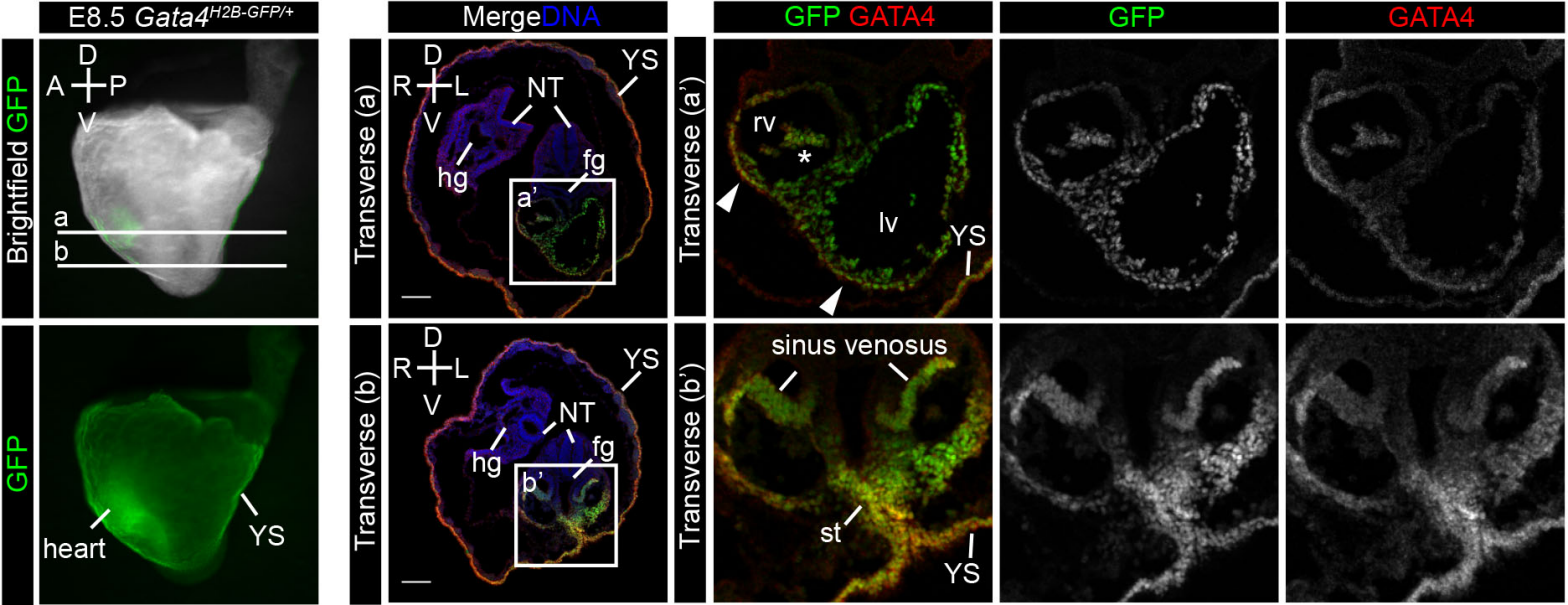
***Gata4^H2B-GFP/+^* reporter expression at E8.5.** Left panels show lateral whole-mount *Gata4^H2B-GFP/+^* embryos at E8.5 prior to fixation with GFP overlaid over bright field or GFP alone. (A,B) Transverse sections of whole-mount stained *Gata4^H2B-GFP/+^* embryos for endogenous GATA4 protein. GFP was visualised directly and DNA was stained with Hoechst at positions indicated on whole-mount embryo. (A,A′) GFP expression is observed throughout the endocardium (inner) and myocardium (outer) layers of the heart and is co-expressed with GATA4 protein (B,B′) and also in the sinus venosus, septum transversum (st) and yolk sac (YS) endoderm. Scale bars: 100 μm. Neural tube (NT), foregut (fg), right (R), left (L), dorsal (D), ventral (V), anterior (A), posterior (P), right ventricle (rv), left ventricle (lv). Arrowheads indicate myocardial wall and asterisk indicates endocardium.

To follow *Gata4^H2B-GFP^* expression at later stages, we first visualised whole-mount heterozygous E8.5 embryos under epi-fluorescence and observed GFP expression in the primitive heart (Fig. 5). To assess tissue-specific expression, embryos were stained for GATA4 protein, cryo-sectioned and imaged by confocal microscopy. At this stage, GFP expression was detected in the right and left ventricle of the primitive heart tube, in both the outer myocardium and the inner endocardium (Fig. 5A). The sinus venosus, precursor of the atria, was also positive for GFP (Fig. 5B). GATA4 protein expression overlapped with GFP in the primitive heart tube, although we noted lower levels of GATA4 protein in the region separating the left and right ventricles. Co-expression of GFP and GATA4 was also observed in the septum transversum mesenchyme of lateral mesoderm origin (Fig. 5B). The exVE derived yolk sac endoderm and anterior midgut co-expressed both GFP and GATA4 while the endoderm of the foregut, posterior midgut and hindgut was negative for both GFP and GATA4 protein at this stage (Fig. 5A,B, Fig. S4).

**Fig. 6.**
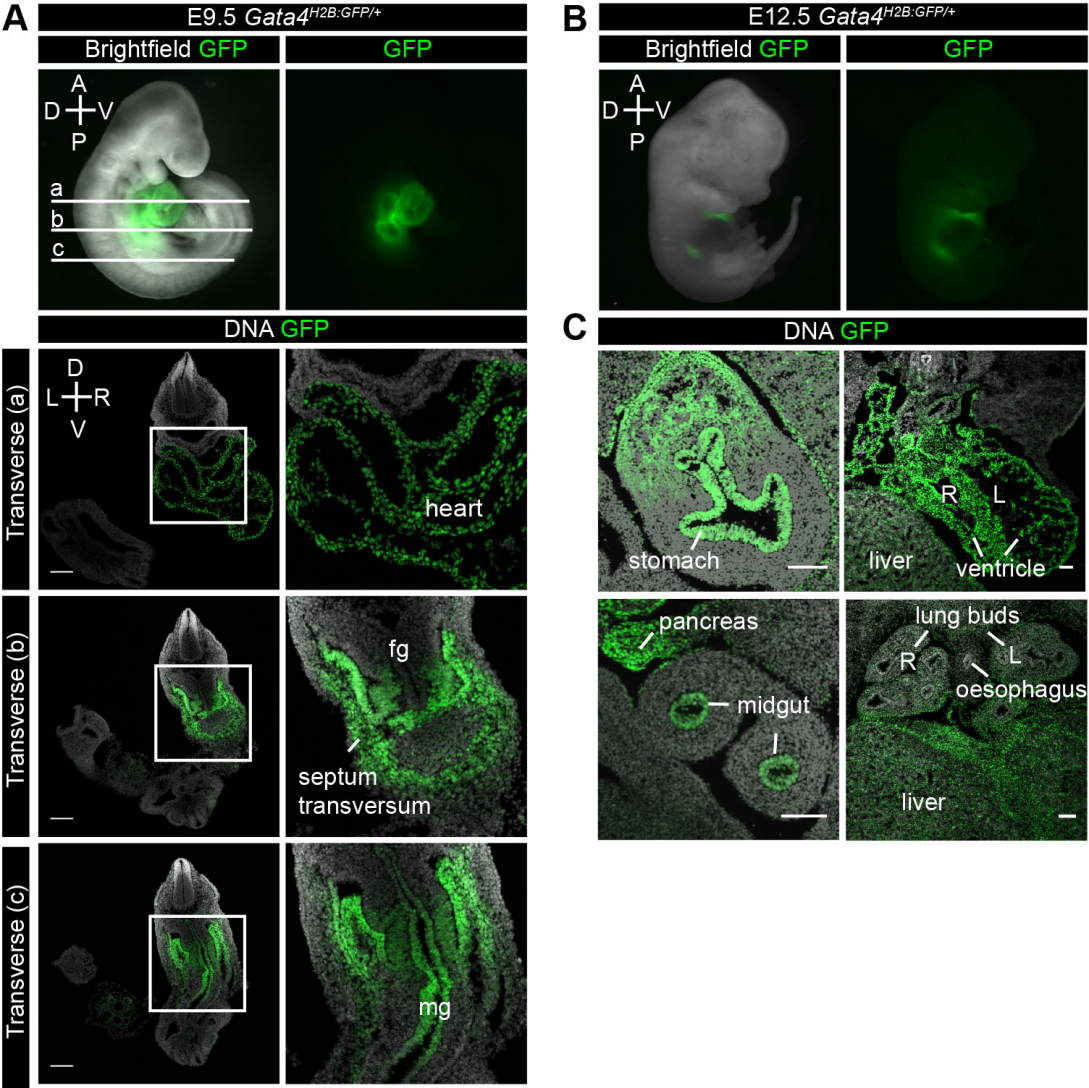
***Gata4^H2B-GFP/+^* reporter expression at midgestation.** (A) Upper panel showing lateral whole-mount immunofluorescence of *Gata4^H2B-GFP/+^* embryos at E9.5. GFP is overlaid over bright field or GFP alone. (a-c) Transverse sections of an E9.5 *Gata4^H2B-GFP/+^* embryo at indicated positions on whole-mount embryo. GFP was visualised directly and DNA was stained with Hoechst (a) GFP continues to be expressed throughout the heart (b) and the septum transversum. (c) GFP is also expressed in the midgut (mg) epithelium at this stage but is absent from the foregut and hindgut. (B) Lateral whole-mount immunofluorescence of *Gata4^H2B-GFP/+^* embryos at E12.5. GFP is overlaid over bright field or GFP alone. (C) Transverse sections of an E12.5 *Gata4^H2B-GFP/+^* embryo. GFP was visualised directly and DNA was stained with Hoechst. GFP expressing cells are present in the stomach epithelium and associated mesenchyme, the liver mesenchyme, the endocardium and myocardium of the heart, pancreas, midgut epithelium and the mesenchyme but not epithelium of the lung buds. Scale bars: 100 μm. Neural tube (NT), foregut (fg), hindgut (hg), right (R), left (L), dorsal (D), ventral (V), anterior (A), posterior (P).

At E9.5 *Gata4^H2B-GFP/+^* embryos showed continued GFP expression within the heart. Sections of E9.5 *Gata4^H2B-GFP/+^* embryos revealed GFP+ cells throughout the endocardium and myocardium of the heart (Fig. 6Aa), in agreement with *Gata4* being expressed throughout cardiac development as a key regulator of cardiogenesis (Arceci et al., 1993; Heikinheimo et al., 1994). In addition, GFP was expressed in the septum transversum at E8.5, but was absent in the adjacent foregut endoderm at E9.5 (Fig. 6Ab). While both the foregut and hindgut endoderm remained negative for GFP, the midgut endoderm robustly expressed *Gata4^H2B-GFP^* at this stage.

At E12.5 GFP expression could be seen in the heart of *Gata4^H2B-GFP/+^* embryos (Fig. 6B). Sections revealed high levels of GFP in the endocardium and myocardium of the heart (Fig. 6C). Tissue-specific expression of *Gata4^H2B-GFP^* was also detected in a number of gut, and gut-associated organs including the midgut endoderm, stomach epithelium and pancreatic bud (Fig. 6C), consistent with known sites of *Gata4* expression (Ketola et al., 2004; Decker et al., 2006; Jacobsen et al., 2002; Heikinheimo et al., 1994). GFP expression was also present in the liver and in the lung bud mesenchyme, but absent in the endoderm-derived lung epithelium (Fig. 6C), as has been shown previously for *Gata4* expression (Ackerman et al., 2007; Jay et al., 2007; Arceci et al., 1993).

### *Gata4^H2B-GFP^* expression in adult mice

**Fig. 7.**
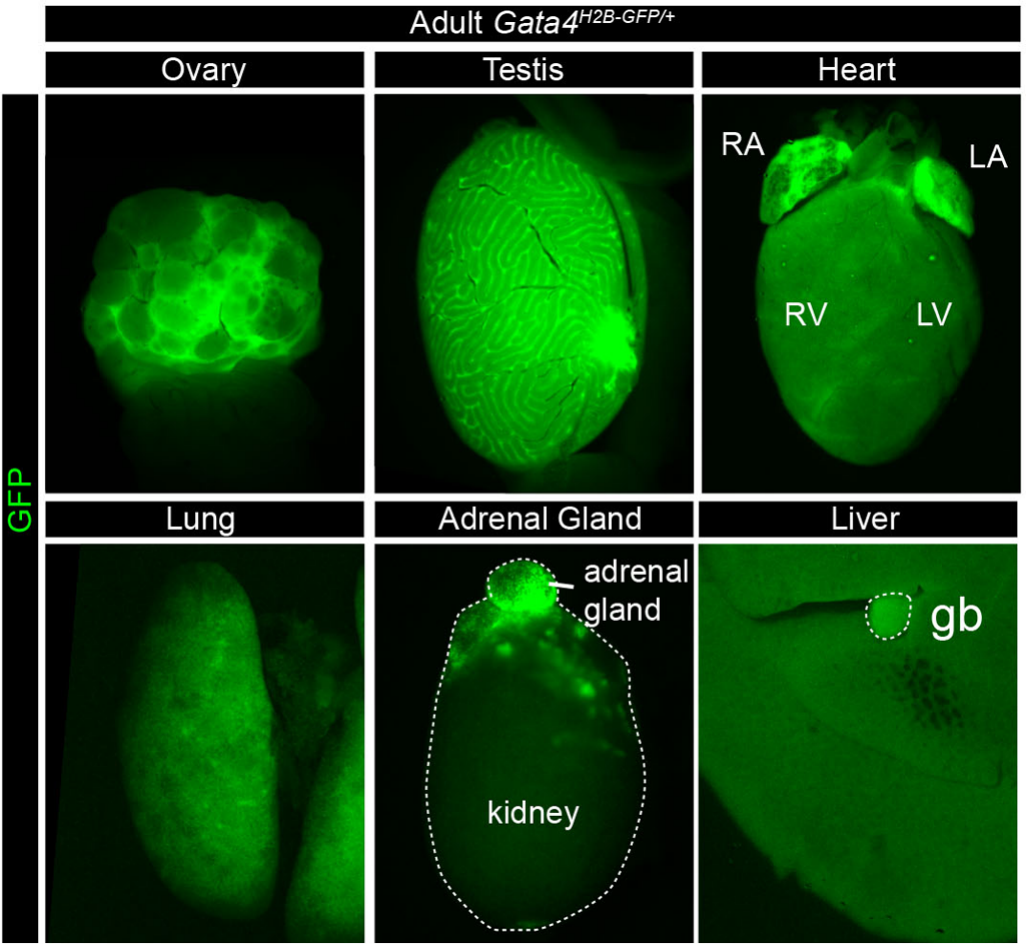
***Gata4^H2B-GFP/+^* reporter expression in adult organs.** Whole-mount immunofluorescence of *Gata4^H2B-GFP/+^* organs from male and female adult mice at 3 months of age. GFP expressing organs include the ovaries, testes, heart, lung, adrenal gland, liver and gall bladder (gb). Right atrium (RA), right ventricle (RV), left ventricle (LV), left atrium (LA).

To assess the expression of the reporter in adult tissues, we visualised whole-mount organs for expression of GFP in both male and female mice at 3 months of age. In *Gata4^H2B-GFP/+^* adults, GFP expression was observed in organs previously reported to express *Gata4* including the heart, lung and liver as noted at E12.5, and in the adrenal gland (Fig. 7) (Arceci et al., 1993; Heikinheimo et al., 1994; Kiiveri et al., 2002). In addition, GFP expression was detected in the gonads, in both the ovary and testis, where *Gata4* is required in granulosa cells for normal ovarian function, and in Sertoli cells for normal testicular function in both foetal and adult mice (Fig. 7) (Kyrönlahti et al., 2011b,a).

## CONCLUSIONS

We have generated a novel *Gata4^H2B-GFP^* reporter and compared its pattern of expression to known sites of *Gata4* mRNA expression and GATA4 protein expression domains. In contrast to previous transgenic reporters that are driven by enhancers or partial rat promoter sequences (Rojas et al., 2005, 2009, 2010; Mazaud Guittot et al., 2007; Pilon et al., 2008; Schachterle et al., 2012), this reporter which is targeted to the endogenous mouse locus retains all *Gata4* cis-regulatory information.

Accordingly, this *Gata4^H2B-GFP^* transcriptional reporter faithfully recapitulated known embryonic *Gata4* expression domains during embryonic development and in adult organs. In endodermal tissues, reporter expression labelled extra-embryonic endoderm and from mid-gestation stages the embryonic midgut endoderm, pancreas and stomach (Plusa et al., 2008; Kurimoto et al., 2006; Arceci et al., 1993; Cai et al., 2008; Heikinheimo et al., 1994; Morrisey et al., 1998; Ketola et al., 2004; Decker et al., 2006; Jacobsen et al., 2002). In mesodermal tissues, reporter expression labelled the cardiac lineage, the lateral plate mesoderm, septum transversum, and pulmonary and hepatic mesenchyme (Ackerman et al., 2007; Jay et al., 2007; Arceci et al., 1993; Nemer and Nemer, 2003; Rojas et al., 2005; Schachterle et al., 2012; Zhao et al., 2008; Heikinheimo et al., 1994). Transcriptional reporter expression strongly correlated with protein levels at pre-implantation embryonic stages, and GFP and GATA4 protein expression were largely co-expressed at post-implantation embryonic stages.

We demonstrated that at pre-implantation stages this *Gata4^H2B-GFP^* reporter is PrE-lineage specific. As *Gata4* is not expressed in uncommitted ICM cells (Plusa et al., 2008; Artus et al., 2011; Kurimoto et al., 2006; Cai et al., 2008) we used it to visualise PrE emergence in live embryos. If combined with spectrally distinct Epi-specific reporter, *Gata4^H2B-GFP^* could be used to study initial specification events as the Epi and PrE lineages emerge from uncommitted ICM progenitors.

At post-implantation stages, *Gata4^H2B-GFP^* robustly labelled both embryonic and extra-embryonic VE cells. Primary defects in the VE cause *Gata4*^−/−^ embryos to arrest between E7.5–E9.0 from either abnormal gastrulation, or later ventral closure defects (Molkentin et al., 1997; Kuo et al., 1997; Narita et al., 1997). *Gata4* clearly plays an important role in VE morphogenesis, but this has not been well studied *in vivo*. Therefore, the *Gata4^H2B-GFP^* reporter allele will be a useful tool to study morphogenetic movements in the VE and interactions with the DE. *Gata4^H2B-GFP^* functions as a null allele so could be used to investigate perturbed functions of *Gata4*-deficient VE cells and probe the primary phenotype seen in *Gata4*^−/−^ embryos (Molkentin et al., 1997; Kuo et al., 1997). In addition, the *Gata4^H2B-GFP^* allele can be Cre-excised to generate *Gata4^FLAG^* allele encoding an *N*-terminally FLAG-tagged GATA4 protein to study GATA4 transcriptional targets and co-factors in tissues of interest.

Another critical role for GATA4 is in cardiogenesis, and we demonstrate that the *Gata4^H2B-GFP^* labels the entire cardiac lineage from gastrulation stages into adulthood, recapitulating endogenous *Gata4* expression (Auda-Boucher et al., 2000; Arceci et al., 1993; Heikinheimo et al., 1994; Schachterle et al., 2012; Rojas et al., 2005; Zhao et al., 2008). Hence, the *Gata4^H2B-GFP^* allele could be used for visualisation and/or FACS isolation of cardiac mesoderm cells at different embryonic stages.

In summary, *Gata4^H2B-GFP^* is a novel mouse line that permits the visualisation of *Gata4* expressing cells at single-cell resolution due to the nuclear-localised fluorescent GFP reporter. This reporter was sufficiently bright to study tissues with time-lapse imaging and at single-cell resolution. As such, this *Gata4^H2B-GFP^* allele will be a useful tool for studying *Gata4* expression and transcriptional regulation in a variety of contexts.

## MATERIAL AND METHODS

### Mice

The *Gata4^H2B-GFP/+^* targeted allele was generated by inserting LoxP-H2B-GFP-4XPloyA-LoxP-FRT-Neo-FRT-3XFLAG cassette into the start codon of the *Gata4* locus through homologous recombination. The targeting construct contained the cassette flanked by a 4.0kb 5′ homologous arm and a 4.0kb 3′ homologous arm in. The construct was linearised and electroporated into 129/SvJ mouse embryonic stem (ES) cells. Positive ES cells were identified by long-range PCR (Roche, Cat. 04829069001) with primer pair P1: 5′-TGGACGTGGACCACTGAGAGTAGG-3′ and P2: 5′-GCTTTAGTCACCGCCTTCTTGGAG-3′ and primer pair P3: 5′-CATGACATCGATTACAAGGATGACG-3′ and P4: 5′-CCTCAGTCTTCAACTCTCTGAACACC-3′. The PCR fragments were further verified by DNA sequencing. The targeted ES cells were microinjected into blastocysts to generate chimeric mice. The male F0 chimeric mice were crossed with Black Swiss females to generate F1 *Gata4^H2B-GFP/+^* mice. The Neo cassette was removed by crossing with FLP recombinase mice (Rodríguez et al., 2000). To generate the *Gata4^FLAG^* mouse line, H2B-GFP was excised by crossing female *Gata4^H2B-GFP/+^* mice with male *Sox2.Cre* mice (Hayashi et al., 2002). *Gata4^H2B-GFP/+^* and *Gata4^FLAG/+^* mice were then maintained on a CD1 outbred background. *Gata4^H2B-GFP^* mice were genotyped using standard GFP primers (Xenopoulos et al., 2015), and primers specific for the *Gata4* wild-type allele (355 bp) P5: 5′-TTTCCTGAGCAAACCAGAGC-3′ and P6: 5′-CGGAGTGGGCACGTAGAC-3′. *Gata4^FLAG^* mice were genotyped using the primers P7: 5′-TTGAGCGAGTTGGGCCTCTCCTCG-3′ and P8: 5′-AGGGCCACCTGCTTCGTAGGCG-3′ to detect the FLAG (490 bp) and wild-type (230 bp) alleles.

### Western blotting

Male *Gata4^FLAG/+^* mice were mated with outbred CD1 female mice. Individual E7.5 embryo littermates were harvested for protein. Single embryos were lysed in 2% SDS and boiled at 95**°**C for 5 min. Protein extracts were applied for western blotting and detected with an anti-FLAG antibody (Sigma-Aldrich, F3165). Total protein lysate from a male *Gata4^FLAG/+^* ear was used as positive control.

### Embryo manipulation and culture

*Gata4^H2B-GFP/+^* males were mated with outbred CD1 female mice. For pre-implantation embryo analysis E3.5–E4.5 blastocysts were flushed from uterine horns using flushing and holding medium (FHM, Millipore) as previously described (Behringer et al., 2014). The zona pellucida of the blastocysts was removed by incubation in acid Tyrode's solution (Sigma-Aldrich) at 37°C for 2 min. For live image analysis, blastocysts were moved into droplets of at least 1 μl of KSOM-AA (Millipore) culture medium per embryo or 10 μM cycloheximide (Sigma-Aldrich) in KSOM-AA, covered with mineral oil (Sigma-Aldrich) in 35 mm glass-bottomed dishes (MatTek). Cultured blastocysts were imaged overnight on a Zeiss LSM880 confocal microscope in a humidified incubation chamber with 5% CO_2_ at 37°C for 16 h and 12 h for cycloheximide treatments. Blastocysts were washed in PBS with 4 mg/ml BSA, prior to fixation in a solution of 4% PFA in PBS (BioRad) for 10 min at room temperature or overnight at 4°C, depending on the anti-GATA4 antibody used (see below). Fixed blastocysts were stored in PBS 4 mg/ml BSA until processing. For post-implantation embryos E5.5–E12.5 embryos were dissected from the maternal decidua in DMEM:F12 medium (Life Technologies). E5.5–E9.5 embryos were washed briefly in PBS and fixed in 4% PFA in PBS for 20 min at room temperature. E12.5 embryos were fixed at 1% PFA in PBS overnight at 4°C. Adult organs were fixed in 4% PFA in PBS for 2 h at room temperature.

### Immunofluorescence of pre-implantation embryos

Pre-implantation embryo immunofluorescence was carried out as previously described (Saiz et al., 2016a). Briefly, fixed blastocysts were washed in PBX; 0.1% Triton X-100 (Sigma-Aldrich) in PBS, permeabilised for 5 min in a solution of 0.5% Triton X-100, 100 mM glycine in PBS and then washed in PBX for 5 min. Embryos were blocked in blocking buffer: 2% horse serum in PBS, for 40 min at room temperature, followed by incubation overnight at 4°C with primary antibodies diluted in blocking buffer (see antibody list below). The next day, embryos were washed three times in PBX (5 min each wash), incubated in blocking buffer for 40 min at room temperature before a 1 h incubation with secondary antibodies at 4°C. Embryos were then washed and incubated for at least 1 h in 5 μg/ml Hoechst in PBS.

For post-implantation stages, fixed embryos were washed in PBX, permeabilised in 0.5% Triton-X in PBS for 15 min and then washed three times in PBX. Embryos were then incubated in blocking buffer, for E5.5–E7.5; 2% Horse serum in PBX, and for >E8.5; 5% horse serum, 0.2% BSA, in PBX, for 2 h at room temperature, followed by incubation overnight at 4°C with primary antibodies diluted in blocking buffer. The next day, embryos were washed extensively in PBX, followed by incubation with secondary antibodies in blocking buffer for 2 h at room temperature, washed in PBX, and incubated with 5 μg/ml Hoechst in PBX to visualise DNA for 2 h to overnight prior to imaging.

Primary antibodies used were rabbit anti-GATA4 (Santa Cruz, sc-9053, 1:100), rabbit anti-NANOG (Reprocell, REC-RCAB0002PF, 1:500), goat anti-GATA4 (Santa Cruz, sc-1237, 1:100, samples fixed overnight at 4°C), goat anti-SOX17 (R&D Systems, AF1924, 1:100), goat anti-GATA6 (R&D Systems, AF1700, 1:100), goat anti-SOX7 (R&D Systems, AF2766, 1:100) and goat anti-BRACHYURY (R&D Systems, AF2085, 1:100). GFP was visualised directly except for when pre-implantation embryos were fixed overnight at 4°C (for use with goat anti-GATA4) when GFP was detected with chicken anti-GFP antibody (Aves, GFP1020, 1:400). Secondary antibodies used in this study were donkey anti-rabbit A647 (Life Technologies, A-315730), donkey anti-goat A568 (Life Technologies, A-11057), goat anti-rabbit A568 (Life Technologies, A-11011) and donkey anti-chicken A488 (Jackson Labs, 703-545-155) all used at 1:500 dilutions.

### Cryo-sectioning of post-implantation embryos

Fixed embryos were washed in PBS and incubated in 10% sucrose for 30 min, followed by incubation in 30% sucrose for 4 h at room temperature. Embryos were then incubated in OCT (Tissue-Tek) overnight at 4°C and cryo-embedded in OCT by freezing on dry ice. Cryo-sections were cut at a 12 μm thickness on a Leica CM3050S cryostat. Cryo-sections that were not stained as whole-mount embryos were washed in PBS then counterstained by incubation in 5 μg/ml Hoechst in PBS for 30 min. Prior to mounting, sections were washed in PBS, and then in water, and mounted in Fluoromount G mounting media (Southern Biotech).

### Image data acquisition

E3.5–E7.5 whole-mount embryos and cryo-sections were imaged on a Zeiss LSM880 laser scanning confocal microscope. Fixed whole-mount embryos were imaged in glass bottomed dishes (MatTek) in PBS (post-implantation) or 5 μg/ml Hoechst in PBS (pre-implantation). Fixed pre-implantation embryos were imaged along the entire z-axis with 1 μm z-steps using EC Plan-Neofluar 40×/1.30 oil immersion objective as described previously (Saiz et al., 2016a). Laser power was measured prior to each imaging session and laser output adjusted to maintain constant power across experiments for each laser line. Accordingly, imaging parameters for each antibody and laser line combination were the same across experiments, to reduce technical variability. For live imaging blastocysts were collected as described and imaged at 2 μm z-steps with a 15 min time-lapse interval. Whole mount embryos E8.5–E12.5 and organs from male and female 3-month-old adult mice were imaged whole-mount on a Leica M165 FC fluorescence stereomicroscope. Raw images were processed in Image J. Time-lapse movies were compiled in Imaris and Quicktime.

### Calculation of half-life in cycloheximide treated embryos

Individual *Gata4^H2B-GFP^* positive PrE cells in time-lapse movies of control (KSOM) and cycloheximide treated embryos were manually tracked in Imaris. High concentrations of cycloheximide are toxic for embryos, therefore we used a relatively low concentration (10 μM) to partially inhibit protein translation, although we still observed increased rates of cell death in cyclohexmide treated embryos. To ensure we only measured H2B-GFP decay, and not the emergence of *Gata4* expressing PrE cells, we only tracked GFP positive cells present at the beginning of the time-lapse movies (∼E4.0). Noise in the raw GFP nuclear fluorescence intensity due to decay in fluorescent signal along the z-axis was not corrected for. In addition, only a few GFP+ cells in cycloheximide treated embryos could be tracked throughout the entire 12 h movie. Shortened tracks were either due to the signal decaying to background levels so that the cell could no longer be identified within the ICM, or apoptosis at earlier time points. Therefore, to normalise for intensity changes within each cell, an exponential decay model for GFP was fitted for individual cell tracks over time. Exponential decay was fitted using Eqn 1. [GFP]_t_ is the fluorescent intensity of GFP at time=t, [GFP]_0_ is the initial fluorescence intensity of GFP, t is time in hours, and k is the decay constant which can also be expressed as the rate of change in log[GFP] as plotted in Fig. S2B. Using Eqn 2 we calculated the half-life (t_1/2_) of H2B-GFP for each individually tracked cell. For values where k→0 then t_1/2_→∞ and skew the mean of t_1/2_, therefore we calculated median as a better measure of central tendency, and the median t_1/2_=5.5 h.
(1)
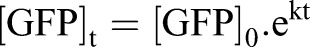

(2)
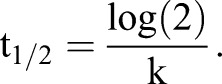


### Quantitative analysis of pre-implantation embryos

Semi-automated 3D nuclear segmentation for cell count staging and quantification of nuclear fluorescence intensity was carried out using MINS, a nuclear-segmentation algorithm, as described previously (Saiz et al., 2016a; Lou et al., 2014). Correction for fluorescence decay along the z-axis was performed by fitting linear regressions to the logarithm of fluorescence values as a function of the z-axis and correcting using an empirical Bayes method, as detailed in (Saiz et al., 2016b). All analysis of fluorescence intensity levels and statistics were performed using R (http://www.r-project.org/). Lineage assignments were determined as follows. Trophectoderm cells were manually scored based on position and morphology. ICM cells were identified as GATA4+PrE or GATA4−ICM cells based on threshold levels of GATA4 protein manually determined from frequency density plots (Fig. S1A). Mitotic cells were excluded from statistical analysis of correlation between GATA4 and GFP fluorescent intensity levels, as H2B-GFP remains bound to chromatin in the absence of nuclear GATA4 protein (Fig. 2B). ICM cell identities were assigned based on relative GATA6 and NANOG levels using a k-means clustering approach as previously described (Saiz et al., 2016b,a), as PrE (GATA6+), EPI (NANOG+), double positive (DP, NANOG+GATA6+) or double negative (DN, NANOG−GATA6−) (Fig. 2C, Fig. S1C).

Primitive endoderm identity was assigned as ICM cells exceeding a manually determined threshold level of GATA4 protein based on frequency density plots of expression levels TE (negative) versus ICM (positive and negative, bimodal). The remaining unassigned ICM cells were classified as ‘epiblast/uncommitted ICM’ as these possibilities could not be distinguished in this experimental regime. Mitotic cells were excluded from statistical analysis of fluorescent intensity levels, as H2B-GFP remains bound to chromatin in the absence of nuclear GATA4 protein.

## Supplementary Material

Supplementary information
