# Supplementary material for: A Gata4 nuclear GFP transcriptional reporter to study endoderm and cardiac development in the mouse
Source: Biol Open. 2018 Dec 15;7(12):bio036517. doi: 10.1242/bio.036517 (PMC6310872; doi:10.1242/bio.036517)
Supplement: Supplementary information [file biolopen-7-036517-s1.pdf]

Parent mating	Offspring genotype		
	Gata4$^{FLAG/FLAG}$	*Gata4*$^{FLAG/+}$	*Gata4*$^{+/+}$
Gata4$^{FLAG/+}$ x *Gata4*$^{FLAG/+}$	7 (35%)	11 (55%)	2 (10%)
Gata4$^{FLAG/FLAG}$ x *Gata4*$^{FLAG/FLAG}$	15 (100%)	-	-

Supplemental Table 1. *Gata4*$^{FLAG/FLAG}$ mice are fertile and viable. Genotype of offspring from *Gata4*$^{FLAG/+}$ and *Gata4*$^{FLAG/FLAG}$ matings

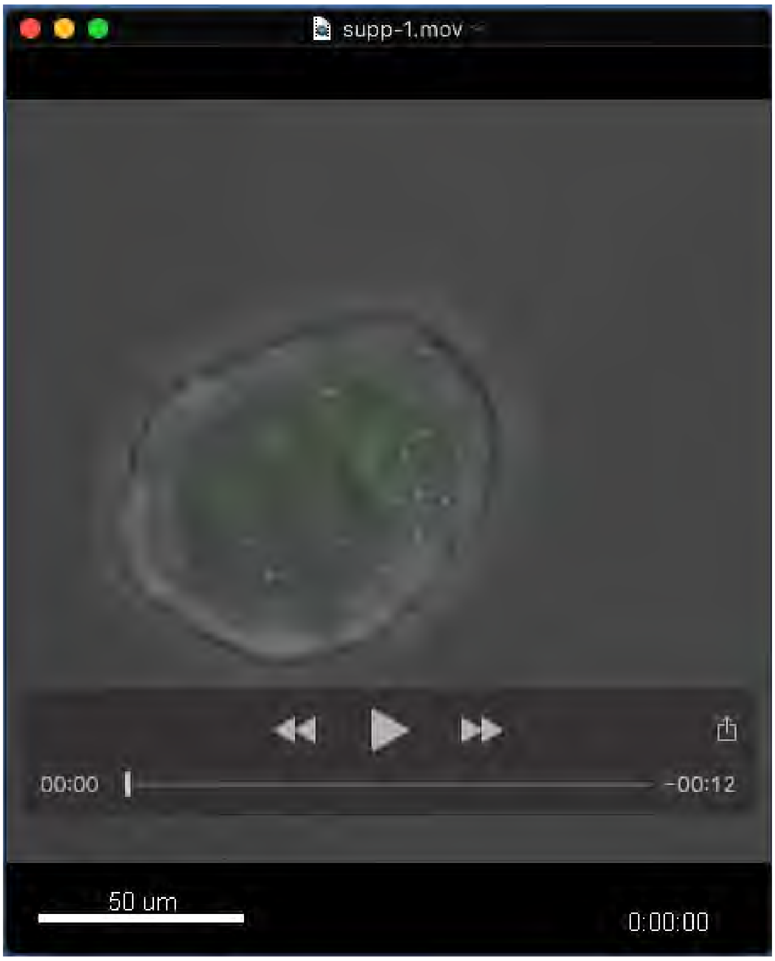

Supplemental Movie 1
Time-lapse confocal microscopy of E3.5 *Gata4*$^{H2B\text{-}GFP/+}$ embryo with GFP (green) overlaid on bright field (BF) and then GFP alone (grey). Scale bar = 50µm. Still images shown in Fig. 1F.

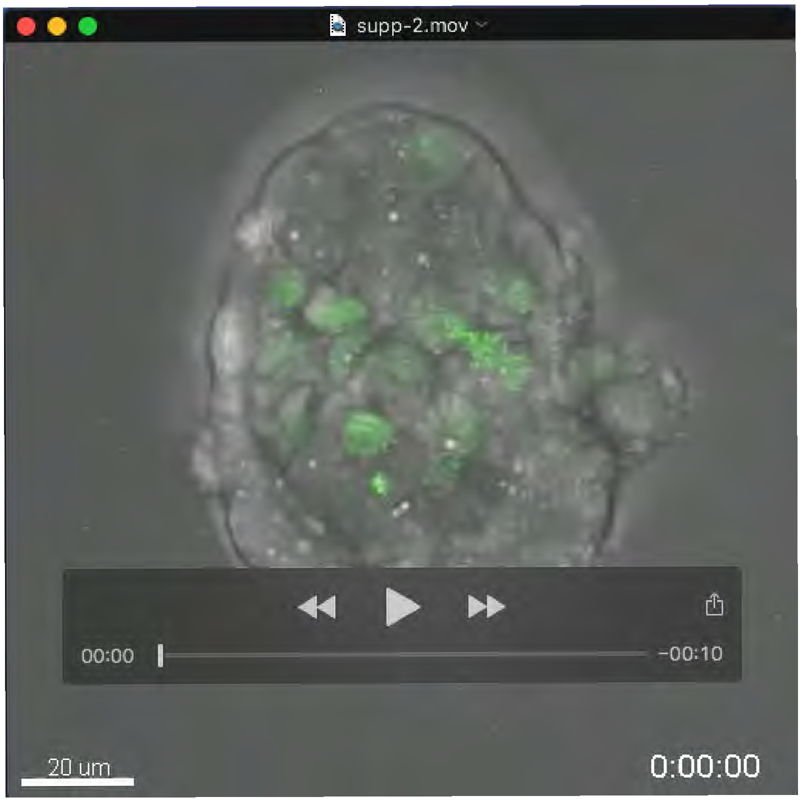

Supplemental Movie 2

Time-lapse confocal microscopy of a control (KSOM) cultured E4.0 *Gata4*$^{H2B\text{-}GFP/+}$ embryo with GFP (green) overlaid on bright field (BF) and then GFP alone (grey). Scale bar = 20μm. Still images and quantification shown in Supplemental Fig. 2.

Biology Open • Supplementary information

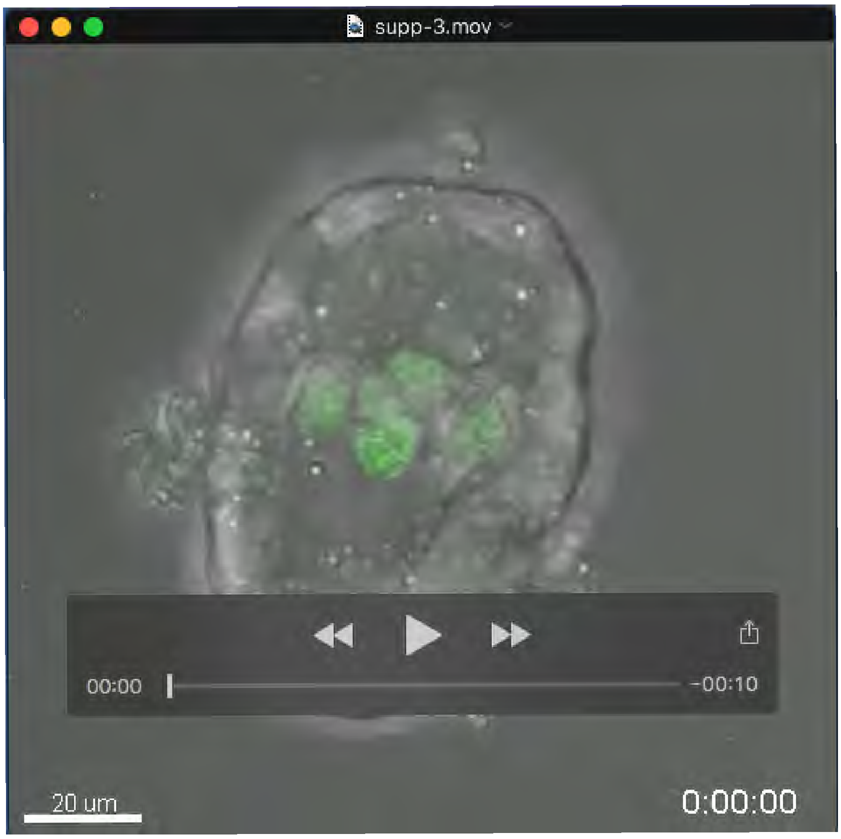

Supplemental Movie 3
Time-lapse confocal microscopy of a cycloheximide (10μM) cultured E4.0 *Gata4*$^{H2B\text{-}GFP/+}$ embryo with GFP (green) overlaid on bright field (BF) and then GFP alone (grey). Scale bar = 20μm. Still images and quantification shown in Supplemental Fig. 2.

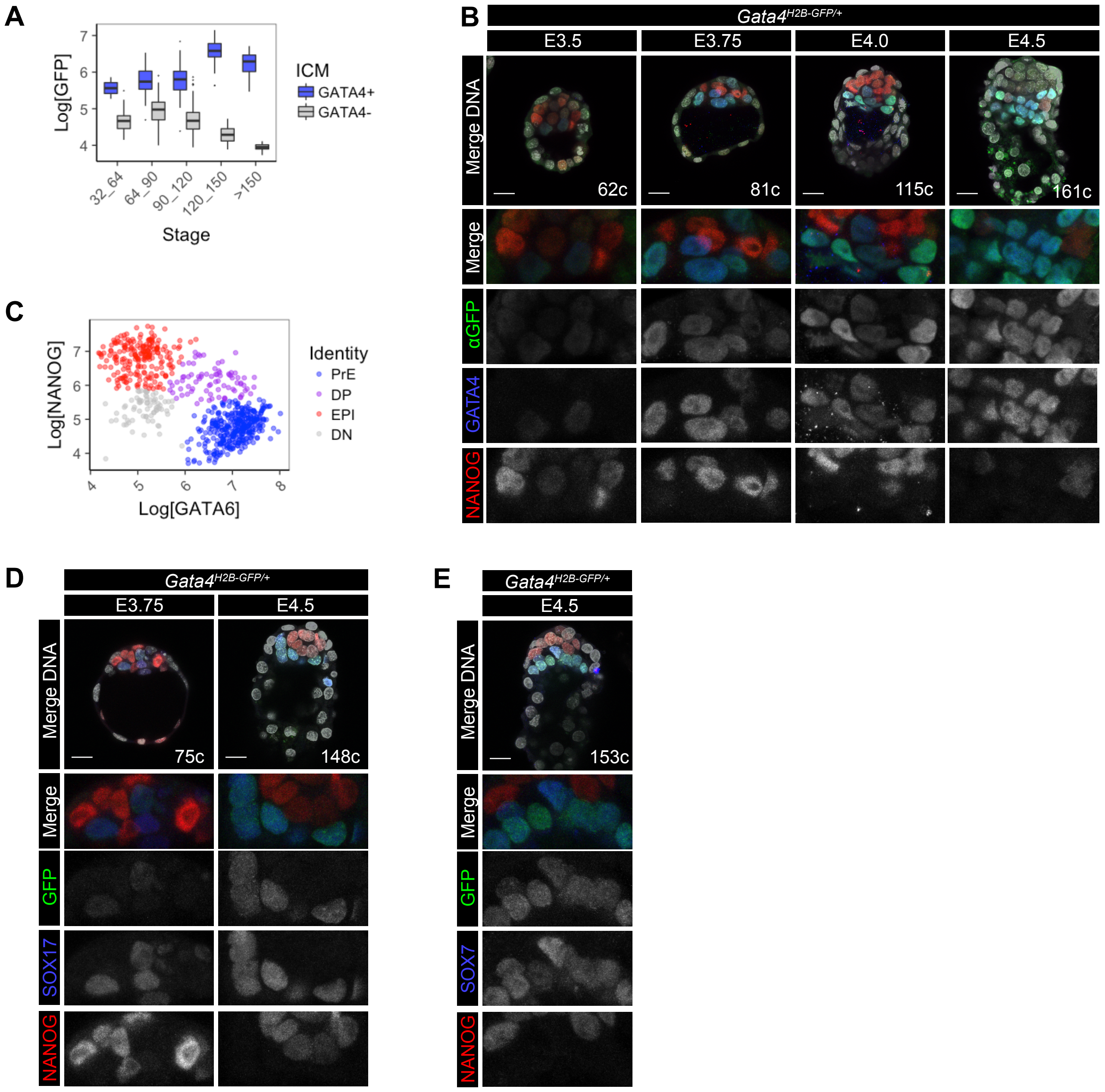

Supplemental Figure 1. *Gata4*$^{H2B\text{-}GFP}$ expression marks primitive endoderm during pre-implantation development. (A) Boxplots showing the logarithmic GFP nuclear fluorescence intensity in GATA4+ primitive endoderm (PrE, blue) or GATA4- (grey) ICM cells in *Gata4*$^{H2B\text{-}GFP/+}$ embryos (n=19) shown in Fig. 2A. **(B)** Representative images showing 10µm maximum intensity projections of confocal z-stack images of fixed *Gata4*$^{H2B\text{-}GFP/+}$ embryos immunostained for GATA4 (goat antibody), anti-GFP and NANOG at E3.5 – E4.5 stages of pre-implantation development. Nuclei were stained with Hoechst. **(C)** Scatter plot showing the k-means clustering to assign ICM lineages based on the relative logarithmic nuclear fluorescence intensity of NANOG and GATA6 for Fig. 2D **(D,E)** Representative images showing 10µm maximum intensity projections of confocal z-stack images of fixed *Gata4*$^{H2B\text{-}GFP/+}$ embryos immunostained for NANOG and the PrE markers SOX17 (D) or SOX7 (E). GFP was visualised directly and nuclei were stained with Hoechst. Embryo stages are also indicated by cell (c) number. Scale bars = 20µm.

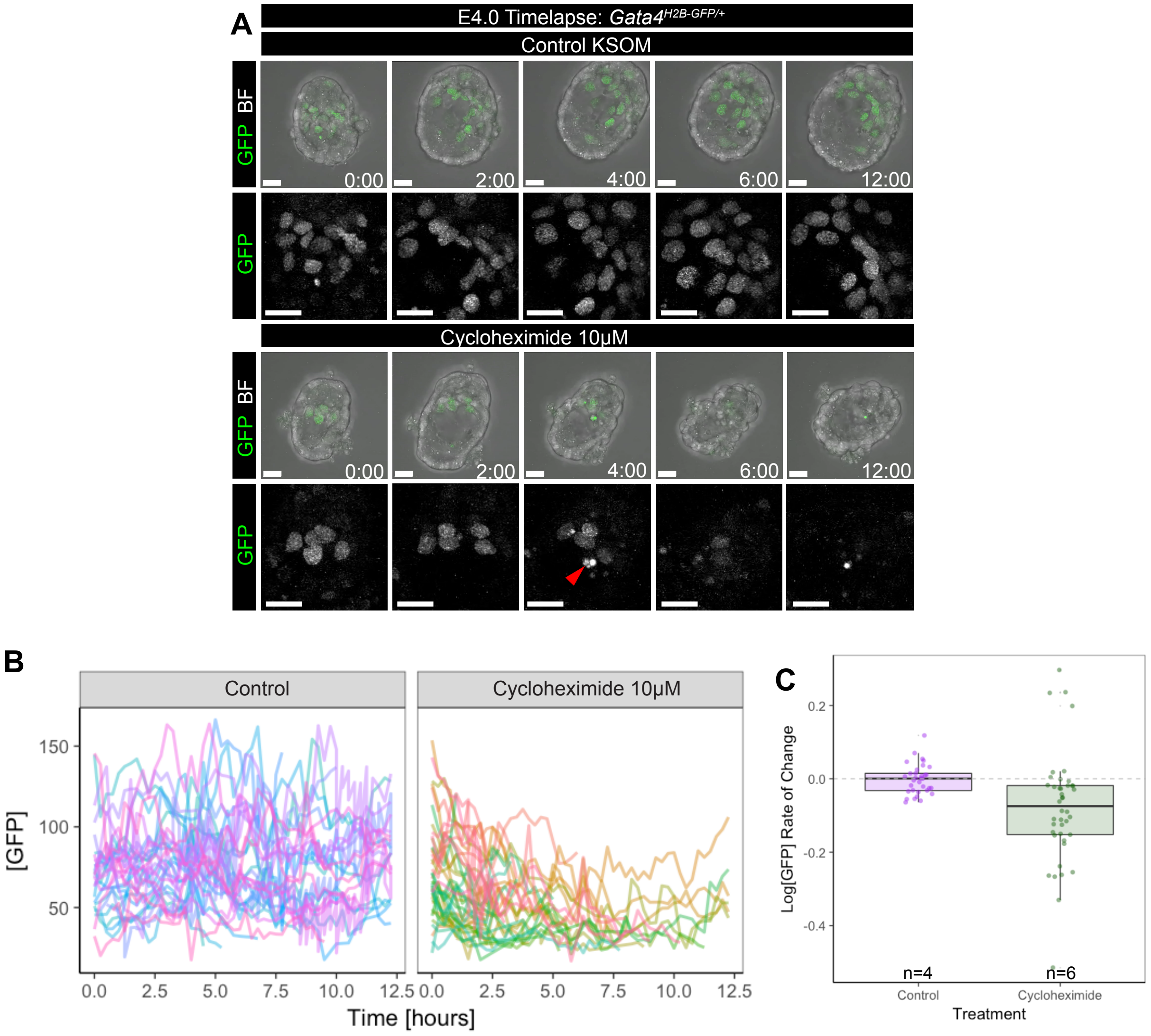

Supplemental Figure 2. Quantitative analysis of *Gata4*$^{H2B\text{-}GFP}$ reporter decay rates using cycloheximide. (A) Still images of time-lapse microscopy of E4.0 *Gata4*$^{H2B\text{-}GFP/+}$ embryos cultured for 12 hours in control (KSOM) or treated with cyclohexmide (CHX, 10µM) to partially inhibit protein translation. GFP overlaid on bright field (BF) or GFP in alone. High rates of apoptosis (red arrowhead) were seen in CHX treated embryos. Scale bars = 20µm. **(B)** Line plots of GFP nuclear fluorescence intensity over time in individually tracked PrE cells from control (n=4) and CHX treated (n=6) embryos. **(C)** Rate of change over time of the logarithmic GFP fluorescence intensity calculated from an exponential decay fit model of each tracked cell. Estimated half-life for CHX treated embryos is 5.5 hours.

Biology Open • Supplementary information

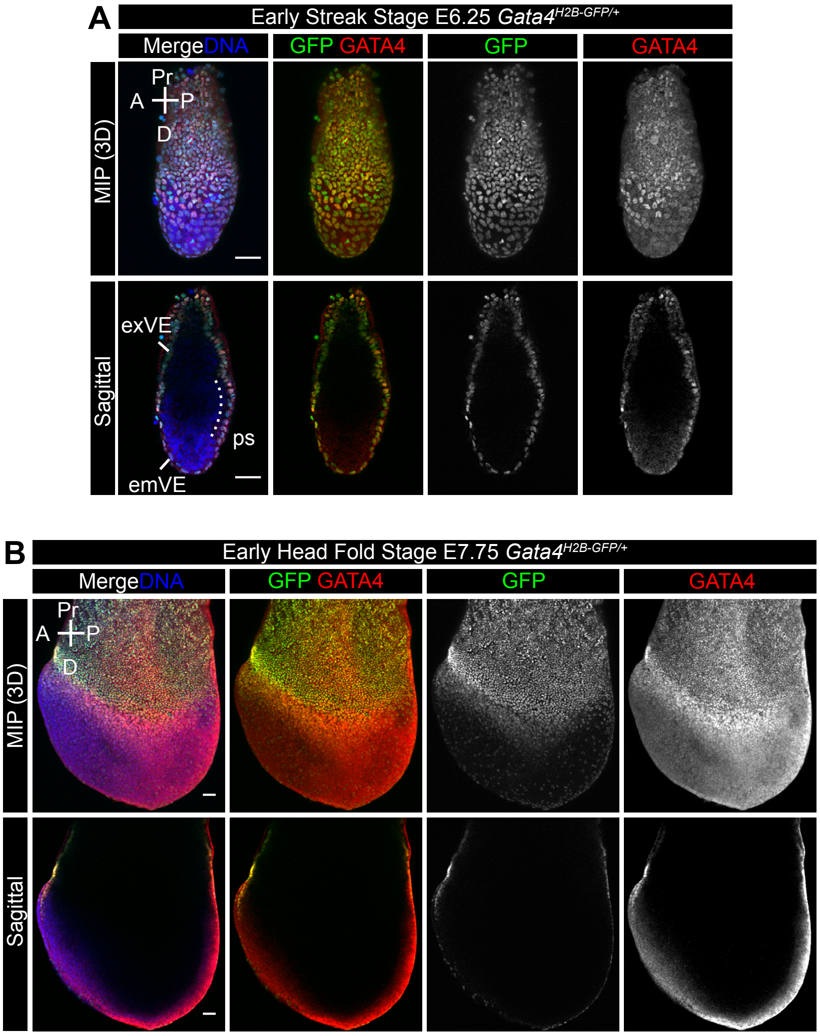

Supplemental Figure 3. *Gata4$^{H2B-GFP/+}$* reporter expression marks the visceral endoderm, cardiac and lateral plate mesoderm at gastrulation stages. (A-B) Representative confocal images of fixed whole-mount *Gata4$^{H2B-GFP/+}$* embryos immunostained for endogenous GATA4 protein at E6.25 **(A)** and E7.75 **(B)** post-implantation stages during gastrulation. Upper panels show maximum intensity projection (MIP) and lower panels show sagittal views of a single optical plane (B). Nuclei are stained with Hoechst and GFP was visualised directly. (A) GFP expression throughout the embryonic and extra-embryonic visceral endoderm (emVE and exVE) and co-localisation with endogenous GATA4 protein. (B) After definitive endoderm intercalation and emVE dispersal, GFP+ emVE cells remain dispersed over the epiblast. GFP+/GATA4 positive cells reside in the mesoderm layer at the proximal-anterior region of the embryo. Upper panels show maximum intensity projection (MIP) and lower panels show sagittal views of a single optical plane. Scale bars = 50µm. Anterior (A), posterior (P), proximal (Pr), distal (D), mesoderm (mes), definitive endoderm (DE).

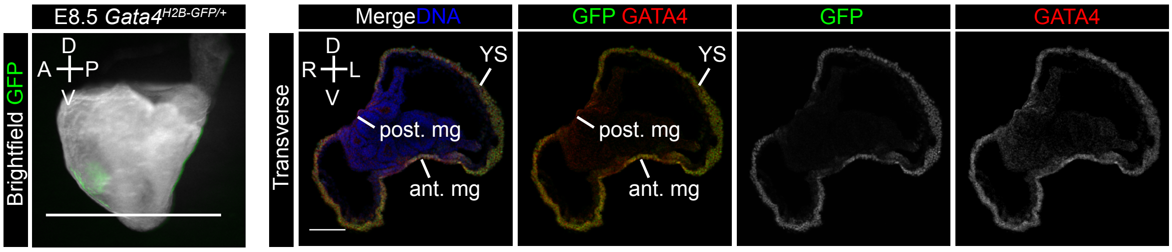

Supplemental Figure 4. *Gata4^H2B-GFP/+^* reporter expression in the midgut at E8.5. Left panel shows lateral whole-mount *Gata4^H2B-GFP/+^* embryos at E8.5 prior to fixation with GFP overlaid over bright field as in Fig. 5. Transverse sections of whole-mount stained *Gata4^H2B-GFP/+^* embryos for endogenous GATA4 protein. GFP was visualised directly and DNA was stained with Hoechst at positions indicated on whole-mount embryo. GFP and GATA4 expression is observed in the yolk sac (YS) and anterior midgut (ant. mg) but absent in the posterior midgut (post. mg). Scale bars = 100μm. right (R), left (L), dorsal (D), ventral (V), anterior (A), posterior (P).

Biology Open • Supplementary information
